# Induction of Transgene Suppression in Plants via External Application of Synthetic dsRNA

**DOI:** 10.3390/ijms20071585

**Published:** 2019-03-29

**Authors:** Alexandra S. Dubrovina, Olga A. Aleynova, Alexander V. Kalachev, Andrey R. Suprun, Zlata V. Ogneva, Konstantin V. Kiselev

**Affiliations:** 1 Laboratory of Biotechnology, Federal Scientific Center of the East Asia Terrestrial Biodiversity, Far Eastern Branch of the Russian Academy of Sciences, Vladivostok 690022, Russia; shumakova_olga91@mail.ru (O.A.A.); 89146508570@yandex.ru (A.R.S.); zlata.v.ogneva@gmail.com (Z.V.O.); kiselev@biosoil.ru (K.V.K.); 2 Laboratory of Embryology, National Scientific Center of Marine Biology, Far Eastern Branch of the Russian Academy of Sciences, Vladivostok 690041, Russia; akalachev@imb.dvo.ru; 3 Far Eastern Federal University, The School of Natural Sciences, Vladivostok 690090, Russia

**Keywords:** dsRNA, transgene, gene silencing, external RNA application, RNA interference, Arabidopsis thaliana

## Abstract

Recent investigations show that exogenously applied small interfering RNAs (siRNA) and long double-stranded RNA (dsRNA) precursors can be taken up and translocated in plants to induce RNA interference (RNAi) in the plant or in its fungal pathogen. The question of whether genes in the plant genome can undergo suppression as a result of exogenous RNA application on plant surface is almost unexplored. This study analyzed whether it is possible to influence transcript levels of transgenes, as more prone sequences to silencing, in *Arabidopsis* genome by direct exogenous application of target long dsRNAs. The data revealed that *in vitro* synthesized dsRNAs designed to target the gene coding regions of enhanced green fluorescent protein (*EGFP*) or neomycin phosphotransferase II (*NPTII*) suppressed their transcript levels in Arabidopsis. The fact that, simple exogenous application of polynucleotides can affect mRNA levels of plant transgenes, opens new opportunities for the development of new scientific techniques and crop improvement strategies.

## 1. Introduction

The increasing human population and discussions along the safety of transgenic plants promote the development of new strategies to regulate plant properties without genomic manipulations. Numerous investigations show that it is possible to switch off or decrease expression of particular genes for the regulation of plant stress tolerance, growth, and other processes via induction of RNA interference (RNAi or gene silencing process) [1]. It is known that RNAi serves for the regulation of various processes important for plants, such as growth and development, stress adaptation, or synthesis of biologically active compounds [2,3]. In the course of RNAi, double-stranded RNAs (dsRNAs) are processed by a ribonuclease into small interfering RNA (siRNA) or microRNA (miRNA). These small RNAs are incorporated in the RNA-induced silencing complex that provides cleavage, destabilization, or hindering translation of any homologous mRNAs [4]. As a result, the mRNAs are degraded, or their efficient translation is prevented. The application of RNAi-based approaches, for gene regulation in plants, requires plant genomic modifications or application of the weakened plant viruses. 

There are new intriguing messages that synthetic dsRNAs or siRNAs, exogenously applied to the plant surface (direct spray application, mechanical inoculation, loading on clay nanosheets, or using materials promoting RNA adhesion), are capable of entering and spreading into plant vascular system and plant cells [5,6,7,8,9] with the following silencing of the targeted essential genes of infected pathogens and the development of local and system resistance against fungal [5,9,10,11] or viral [6,7,12,13] infections. There is also evidence for processing the external dsRNAs into siRNAs, and induction of RNAi-mediated silencing of the targeted pathogen genes [5,6,7,12,14]. Since RNA molecules can be up-taken and translocated into plant tissues, active studies in this direction are extremely urgent, not only for confirmation and detailed investigation of these facts, but also for investigating the possibility of regulating the expression of plant endogenous genes via this approach. Intriguingly, the patent by Sammons et al. [15] exemplified the possibility of down-regulating transcript levels of plant endogenous genes encoding for herbicide resistance using direct exogenous applications of dsRNA, siRNA, ssRNA, or even DNA molecules. Numata et al. [16] indicated that spraying *Arabidopsis* with synthetic siRNAs in a complex with a protein carrier induced local loss of anthocyanin pigmentation, probably, due to the silencing of a chalcone synthase gene. 

A number of studies provided that plant transgenes are more susceptible to silencing than endogenes [17,18,19,20]. Transgenes usually do not contain introns and 5’/3’-UTRs, which have been shown to contribute to the expression stability of plant endogenes and suppression of RNA silencing [18,19,21]. In addition, transgenes are usually under the control of strong promoters, ensuring a high level of expression and, thus, elevating the possible production of aberrant mRNAs. Aberrant mRNAs have been shown to be perceived by plant RNAi machinery and transcribe into secondary dsRNAs by RNA-directed RNA polymerase 6 (RDR6) leading to amplification of the transgene silencing [22]. The secondary dsRNAs are then converted into secondary siRNAs that map upstream and downstream the primary siRNAs [18]. Therefore, it would be interesting to analyze the influence of external RNAs on transgene activity first. The available studies reported that they failed to regulate the fluorescence of GFP and YFP in transgenic plants by direct siRNA application (e.g. wiping, spraying, injection) without using additional techniques, i.e., a protein carrier or high-pressure spraying [16,23]. Dalakouras et al. [23] reported that spraying *GFP*-transgenic *Nicotiana benthamiana* with synthetic *GFP*-siRNA solutions, under high pressure, using a conventional compressor and an air brush pistol, led to silencing of the GFP fluorescence. The effect was not observed when high pressure was not applied. Numata et al. [16] found that infiltration of *in vitro* synthesized *YFP*-siRNA in a complex with carrier peptide into leaves of *A. thaliana* and poplar resulted in lowering the YFP protein levels and fluorescence observed by confocal laser scanning microscopy. In our study, we aimed to investigate whether it is possible to influence the expression of plant transgenes by simple application of long dsRNA solutions without using additional promoting agents or techniques.

## 2. Results

**Figure 1:**
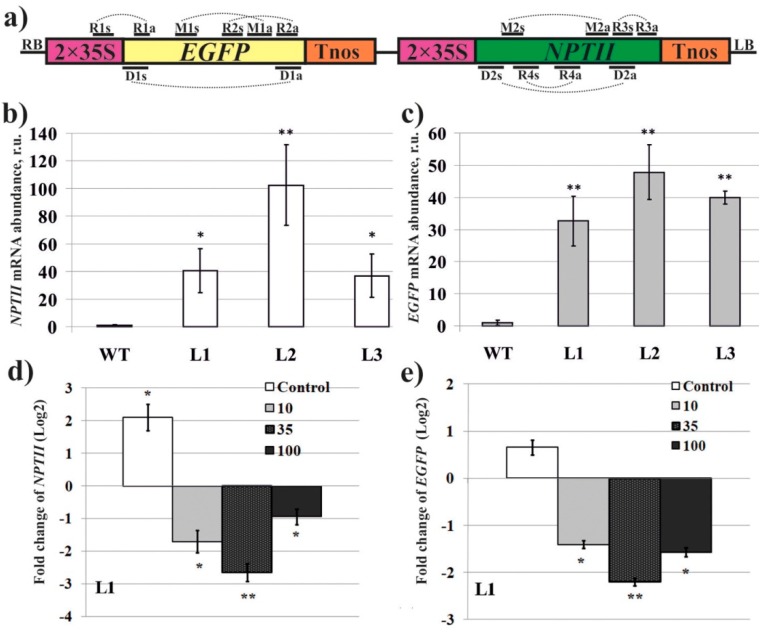
Characterization of transformed *A. thaliana* lines, and investigation of the dsRNA concentration effect. (**a**) Schematic representation of the T-DNA region from the pZP-RCS2-*EGFP*-*NPTII* vector [24]. 2x35S—the double 35S promoter of the cauliflower mosaic virus (CaMV); *EGFP*—the enhanced green fluorescent protein (*EGFP*) gene; *NPTII*—the neomycin phosphotransferase II (*NPTII*) gene; Tnos—nopaline synthase terminator. D1s, D1a, D2s, D2a—primers for dsRNA synthesis; R1s, R1a, R3s, R3a—primers for qRT-PCR estimation of transgene expression after dsRNA treatments; R2s, R2a, R4s, R4a—primers designed to align inside the transgene fragments that have been used for synthesis of the corresponding dsRNAs; M1s, M1a, M2s, M2a—primers for bisulfite DNA sequencing. (**b**,**c**) Quantification the *NPTII* and *EGFP* mRNAs in the untreated transgenic *A. thaliana*. RNA was extracted from the wild type (WT) and transformed *A. thaliana* lines (L1, L2, L3). (**d**,**e**) Quantification the *NPTII* and *EGFP* mRNAs (log2 fold change) in L1 of *A. thaliana* in response to external application of *NPTII*-dsRNA, and *EGFP*-dsRNA 7 days post-treatment, respectively. 10, 35, 100—the synthesized *EGFP*-dsRNA and *NPTII*-dsRNA were diluted in water to concentrations of 0.1, 0.35, and 1 µg/µL (100 µL per plant). Control—sterile filtered water. qRT-PCR data are presented as mean ± SE. *, **—significantly different from the untreated plants at *P* ≤ 0.05 and 0.01, respectively, according to the Student’s *t*-test.

To create transgenic *Arabidopsis*, we introduced pZP-RCS2 [24], bearing the neomycin phosphotransferase II (*NPTII*), and the enhanced green fluorescent protein (*EGFP*) genes under the control of the double CaMV 35S promoters (Figure 1a) into *Agrobacterium tumefaciens* and transformed *A. thaliana* by floral dip [25]. Three representative independent T_3_ homozygous lines with high mRNA levels of *EGFP* and *NPTII* were chosen for detailed analyses. The quantification of the transgene transcript levels in *A. thaliana,* performed by quantitative real-time RT-PCR (qRT-PCR), showed that the transgenic lines displayed high levels of *NPTII* and *EGFP* expression (Figure 1b,c). Then, we used PCR and *in vitro* transcription protocol to produce dsRNA molecules of the *EGFP* and the *NPTII* genes. The complete *EGFP* (720 bp) and a large fragment of the *NPTII* (599 bp out of 798 bp) genes were amplified. The obtained PCR products, containing T7 promoters at both ends, were used as templates for *in vitro* transcription. For external applications, the synthesized *EGFP*-dsRNA and *NPTII*-dsRNA were diluted in water to final concentrations of 0.1, 0.35, and 1 µg/µL. The dsRNAs (100 µL of each dsRNA per individual plant, i.e. 10, 35, and 100 µg) were applied on the surface of four-week-old *Arabidopsis* by spreading with sterile individual soft brushes. The analysis of concentration effect was performed on the L1 line of *A. thaliana* and analyzed 7 days post-treatment, indicating that 0.35 µg/µL, resulted in the highest silencing efficiency (Figure 1d,e). Therefore, this concentration was chosen for the following experiments where we studied whether simple exogenous applications of the *NPTII*-dsRNA and *EGFP-*dsRNA on plant surface of the three transgenic lines L1, L2, and L3 could lead to changes in transcript levels of the transgenes in comparison with the water-treated control plants 1, 7, and 14 days post-treatment.

**Figure 2:**
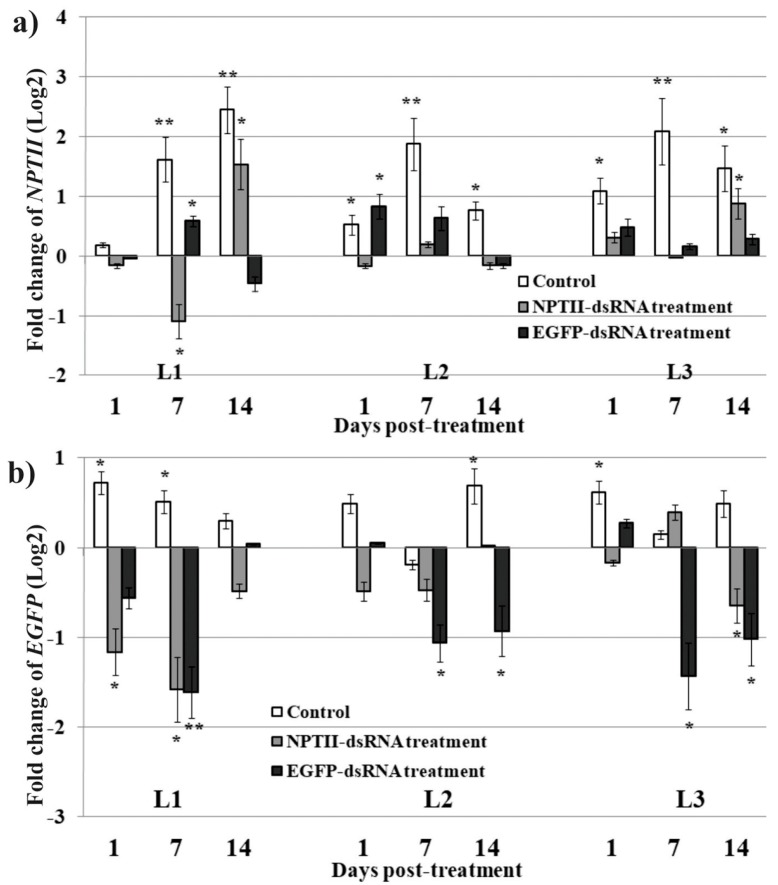
dsRNA-induced *EGFP* and *NPTII* transgene suppression in four-week-old *Arabidopsis thaliana*. The expression levels (log2 fold change) of the *NPTII* (**a**) and *EGFP* (**b**) in response to external application of sterile filtered water (Control), *NPTII*-dsRNA, and *EGFP*-dsRNAs relative to *NPTII* and *EGFP* levels in the plants before treatments. The dsRNA were diluted in water to 0.35 µg/µL (100 µL per plant). The RNA was isolated before, 1, 7, and 14 days post-treatment from an individual plant of each line per treatment (four independent experiments). qRT-PCR data are presented as mean ± SE. *, **—significantly different from the untreated plants at *P* ≤ 0.05 and 0.01, respectively, according to the Student’s *t*-test.

qRT-PCR showed that the *NPTII* transcript levels increased in plants treated with sterile water during cultivation compared with the plants before treatments (Figure 2a). However, the elevation in *NPTII* transcript levels was lower or was not observed in plants treated with external *NPTII*-dsRNA (Figure 2a). As for *EGFP* expression, plants that received *EGFP*-dsRNA exhibited down-regulation or repression of *EGFP* expression after the application of the exogenous *EGFP*-dsRNA, while water-treated plants exhibited either, an increase, or a weak decrease, in *EGFP* expression (Figure 2b).

**Figure 3:**
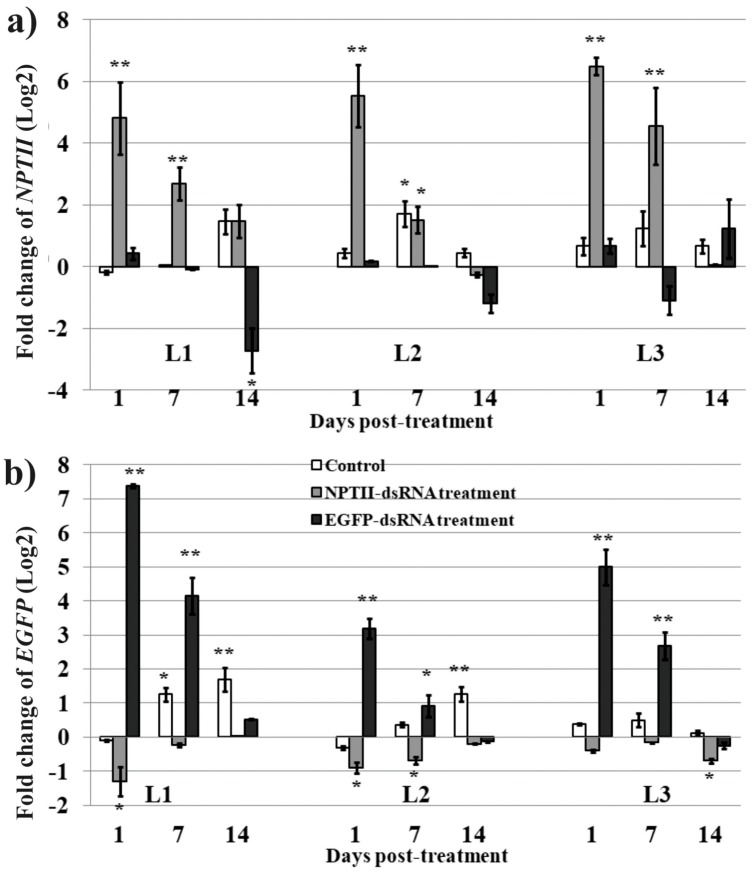
The analysis of *NPTII* (**a**) and *EGFP* (**b**) transgene transcript levels (log2 fold change) using primers designed to align inside the *NPTII* and *EGFP* transgene fragments, which have been used for synthesis of the corresponding dsRNAs, in the dsRNA-treated four-week-old *Arabidopsis thaliana* relative to that in the plants before treatments. One plant of the L1, L2, and L3 lines for each treatment was treated with *NPTII*-dsRNA, *EGFP*-dsRNAs or filtered sterile water (Control). The dsRNA were diluted in water to 0.35 µg/µL (100 µL per plant). In an experiment, the RNA was isolated before, 1, 7, and 14 days post-treatment from each plant (three independent experiments). qRT-PCR data are presented as mean ± SE. *, **—significantly different from the plants before treatment at *P* ≤ 0.05 and 0.01, respectively, according to the Student’s *t*-test.

We also analyzed *NPTII* and *EGFP* expression using primers (Figure 1a; Table S1), designed to align inside the *NPTII* and *EGFP* transgene fragments, that have been used for synthesis of the corresponding dsRNAs (Figure 3a,b). Plants that have been treated with dsRNAs, exhibited dramatically high *NPTII* or *EGFP* levels, which gradually decreased with time of cultivation (Figure 3a,b). The data indicate that the synthetic *NPTII* and *EGFP* dsRNAs have been purified with the total RNA and transcribed into cDNA during reverse transcription. Thus, the *NPTII*- and *EGFP*-dsRNAs were stable for at least 7 days of cultivation and were not completely removed from the treated leaves during this time (Figure 3a,b). Figure 3 shows suppression of *NPTII* in plants that received *EGFP*-dsRNA, and *EGFP* in plants that received *NPTII*-dsRNA, respectively (Figure 3a,b). 

**Figure 4:**
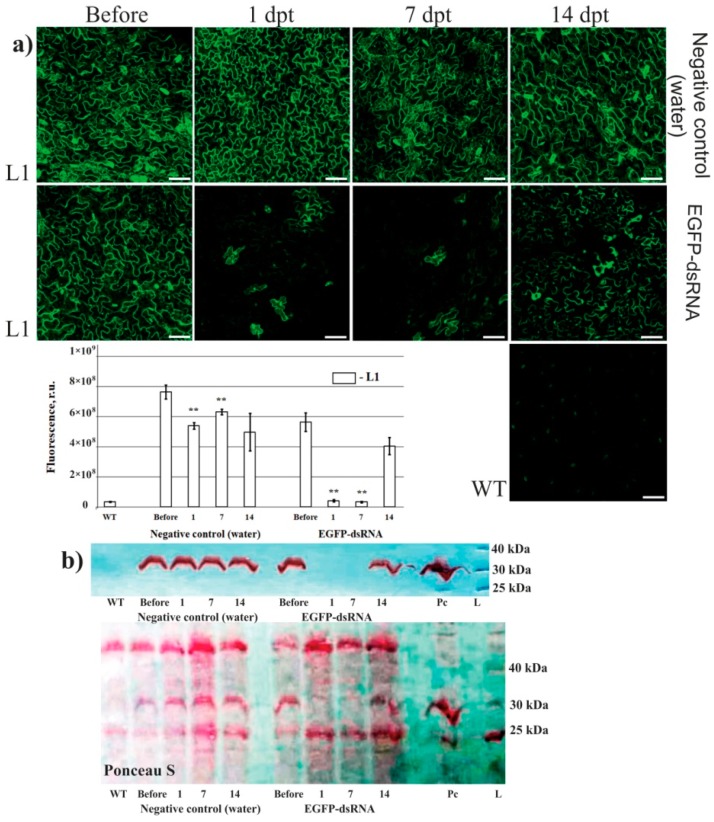
The effect of external dsRNA application on the *EGFP* fluorescence and *EGFP* protein levels in four-week-old *Arabidopsis* plants of the L1 line. *EGFP* silencing efficiency was visualized 1, 7, and 14 days post-treatment of L1 rosettes using confocal microscopy (**a**) and western blotting (**b**). Pc—total protein purified from *E. coli* Rosetta(DE3)pLysS transformed with pET28a-EGFP. Values are the mean ± SE. ** ‒ significantly different from the negative control at *P* ≤ 0.01, respectively, according to the Student’s *t*-test.

Confocal microscopy was used to analyze EGFP fluorescence in L1 of *A. thaliana* and showed that EGFP fluorescence sharply decreased 1 and 7 days post-treatment with the EGFP dsRNA in the L1 line (Figure 4a). The data are in agreement with the *EGFP* gene expression analysis, indicating a large decrease in *EGFP* transcript levels 1 and 7 days post-treatment (Figure 2b). Western blotting showed a sharp decline in EGFP protein level 1 and 7 days post-treatment in L1 (Figure 4b). The results indicate that transgene suppression occurred not only at mRNA but also protein level.

**Figure 5:**
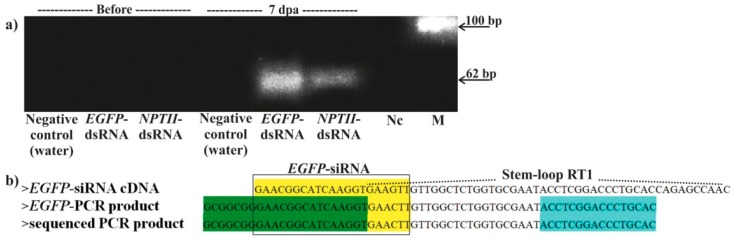
Detection of *EGFP*-siRNA (NCBI siRNA database acession: Pr008640320 or Pr120179) in the dsRNA-treated and water-treated A. thaliana L1 leaves using stem-loop RT-PCR before treatment and 7 days post application. (**a**) Agarose gel electrophoresis of the stem-loop RT-PCR products. M—low molecular weight DNA ladder; Nc—negative control (RT-PCR mixture without RNA). (**b**) Sequences of the cloned RT-PCR product and predicted stem-loop structure of the target *EGFP*-siRNA (sense strand) with primer positions. Dashed lines depict position of the stem-loop RT primer; highlighted in green and blue are the forward and reverse primer positions for stem-loop RT-PCR; highlighted in yellow is the *EGFP*-siRNA sense strand.

To determine whether *EGFP*-siRNAs accumulate in the *A. thaliana* L1 plants, we performed stem-loop RT-PCR as described [26] for small RNA fractions purified from the dsRNA-treated, and water-treated, *A. thaliana* leaves. This method was originally applied for miRNA detection and then successfully used for siRNA detection in plant tissues [7,8,12]. We used as the target siRNA one of the *EGFP*-encoding siRNAs (NCBI GB accession: Pr008640320, Pr120179) with a typical structure and with a documented *EGFP* silencing efficiency [27,28]. Stem-loop RT-PCR followed by cloning and sequencing of the RT-PCR products revealed the presence of the target *EGFP*-siRNA in the leaves treated with the *EGFP*-dsRNA, while the siRNA was not detected in the plants before treatment and in the control water-treated plant (Figure 5). The target *EGFP*-siRNA was also detected in the leaves treated with *NPTII*-dsRNA, though at a much lower level than after treatment with *EGFP*-dsRNA (Figure 5). 

**Figure 6:**
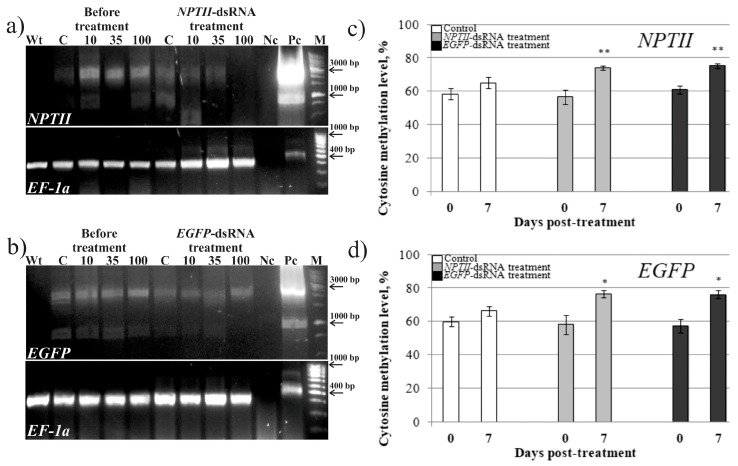
Detection of the read-through transcripts, including both the *EGFP* and *NPTII* sequences, and analysis of the total cytosine DNA methylation, within the *NPTII* and *EGFP* transgenes in the transgenic L1 line of *A. thaliana,* before and 7 days after foliar application of dsRNAs or filtered sterile water. (**a**,**b**) Agarose gel electrophoresis of the RT-PCR products. C—control treatment (sterile filtered water). 10, 35, 100—treatment with the dsRNAs diluted in water to concentrations of 0.1, 0.35, and 1 µg/µL (100 µL per plant). M—low molecular weight DNA ladder; Nc—negative control (RT-PCR mixture without RNA); Pc—positive control (pZP-RCS2 plasmid). (**c**,**d**) Bisulfite sequencing of the DNA purified from the dsRNA- and water-treated plants. The dsRNAs were diluted in water to 0.35 µg/µL (100 µL per plant). The data are presented as mean ± SE. Significant differences at *—*P* < 0.05 and **—*P* < 0.01 versus values of cytosine methylation before treatment according to the Student’s *t*-test. The total number of cytosines was regarded as 100% in each DNA region.

In case the *EGFP*-siRNAs were generated by transitive silencing along the transgene sequence after treatment with the *NPTII*-dsRNA, the read-through transcripts, that include both *NPTII* and *EGFP* sequences should be generated in the L1 transgenic plants. Therefore, we analyzed whether the readthrough transcripts, including both the *NPTII* and *EGFP* sequences, are accumulated (Figure 6a,b; Figure S1; Table S1). For RT-PCR, we used the cDNAs obtained from the transgenic *A. thaliana* L1 plants, treated with the *NPTII*- and *EGFP*-dsRNAs. The RT-PCR analysis (Figure 6a,b) revealed that there was no amplification in the cDNA probes of the wild-type *A. thaliana* plants, while a clear amplification product of 2–2.5 kb was detected in the cDNA probes from the transgenic plants and for the positive control (pZP-RCS2 plasmid). DNA sequencing revealed that the amplification product of 2.276 kb contained coding sequences of both transgenes and their regulatory elements (Figure S1). The RT-PCR analysis also indicated that the abundance of the transgene read-through transcripts was lowered after the dsRNA treatments but not after control water treatments (Figure 6a,b).

When analyzing transgene transcription after dsRNA treatment, we noted that the *NPTII* transcript level was lowered in *A. thaliana* treated with the *EGFP*-dsRNA (Figure 2a). Similarly, the *EGFP* transcript level was down-regulated in the plants treated with *NPTII*-dsRNA (Figure 2b). In case transitivity and silencing amplification occur, the reduction of *NPTII* mRNA levels by application of *EGFP*-dsRNA, and vice versa, can be explained by the secondary siRNA formation. It is also possible that the application of the *NPTII-* or *EGFP*-dsRNAs to individual plants could affect expression of the transgenes due to induction and spreading of DNA methylation at the T-DNA region bearing both transgenes. To examine this hypothesis, we analyzed cytosine DNA methylation levels before and after treatment of *A. thaliana* L1, with *EGFP*-and *NPTII*-dsRNAs, using bisulfite sequencing of the dsRNA-targeted transgene regions (Figure 6c,d; Table 1). The data revealed that the extent of DNA methylation considerably increased after the dsRNA treatments. 

The analysis of *NPTII* and *EGFP* transgene cytosine methylation at different contexts (CG, CHG and CHH) showed that the dsRNA treatments increased methylation in all sequence contexts, while control water treatment did not essentially change methylation status (Table 1). The most pronounced effect for the dsRNA treatments was observed for CHG and CHH methylation elevating it by 1.2-1.3 and 1.3-1.5-fold, respectively. Methylation status at CG positions was considerably affected only for *NPTII* after *NPTII*-dsRNA treatment. 

## 3. Discussion

In this study, the transgenic adult plants of *A. thaliana,* overexpressing the *NPTII* and *EGFP,* have been treated with long dsRNAs targeting these transgenes. Taken together, the results show that the exogenous application of dsRNA spread with soft brushes suppressed *NPTII* and *EGFP* transcript levels in *A. thaliana*. The current data also indicated that applying 35 µg of the transgene-encoding dsRNA per four-week-old plant is the most efficient amount, due to the combination of effectiveness and lower cost of synthesis. Thus, simple foliar applications of dsRNAs complimentary to commonly used transgenes lead to their repression. The detected dsRNA-induced down-regulation of transgene transcript levels suggests that the exogenously applied dsRNAs were uptaken by plants, and initiated RNAi-mediated silencing of the target mRNA transcripts. In our study, the detection of *EGFP*-derived siRNA in the dsRNA-treated plants, indicated that the *EGFP*-encoding dsRNAs were processed into siRNAs, and the observed transgene silencing effect was based on RNAi-mediated silencing. The detection of the *EGFP*-derived siRNA after *NPTII*-dsRNA treatment suggests that *NPTII* silencing was accompanied by the phenomenon of transitivity [18,19]. Transgenes have been shown to be more prone for transitivity, which is the spreading of silencing beyond the initial target site. Transgenes are usually under the control of strong promoters, ensuring a high level of expression and, thus, elevating possible production of aberrant mRNAs, e.g., truncated and/or read-through transcripts, leading to the amplification of the transgene silencing [18,19,22]. In our study, we detected transgene read-through transcripts, including both the *NPTII* and *EGFP* coding sequences and the regulatory elements, in all the dsRNA-treated and non-treated L1 transgenic *A. thaliana* plants. According to the data obtained, the silencing transitivity occurred before and after treatments in the transgenic *A. thaliana* plants and was enhanced after the dsRNA treatments. Surprisingly, we also noted that the *NPTII* mRNA level was suppressed in *A. thaliana* treated with the *EGFP*-dsRNA (Figure 2a). Similarly, the *EGFP* mRNA level was down-regulated in the plants treated with *NPTII*-dsRNA (Figure 2b). If transitivity occurs, the reduction of *NPTII* mRNA levels by the application of *EGFP*-dsRNA, and vice versa, can be explained by secondary siRNA formation. Further experiments, including small RNA sequencing, are needed to examine the whole diversity of the transgene-siRNAs generated after external dsRNA application.

In our study, we also found that the level of cytosine DNA methylation considerably increased after the dsRNA treatments at the dsRNA-targeted protein-coding regions of the transgenes. RNA-directed DNA methylation (RdDM), a process where siRNAs induce *de novo* DNA methylation of the target homologous DNA sequences, and the DNA sequences upstream/downstream of the target sites [19,29,30], is supposed to be responsible for methylation induced at the protein-coding regions of post-transcriptionally silenced genes [31,32]. Methylation at the gene coding regions was also presumed to contribute to PTGS reinforcement. In our study, DNA methylation at the coding sequences of the *NPTII* and *EGFP* transgenes homologous to the applied dsRNAs could reflect the influence of 24nt siRNAs, a RdDM “footprint”, arising as a byproduct of unprecise dicing. Therefore, it is possible that the application of the *NPTII-* or *EGFP*-encoding dsRNAs could promote transgene mRNA degradation and also affect transcription of the transgenes and/or heterochromatin formation, due to the induction and spreading of DNA methylation at the T-DNA region bearing both transgenes. Although only sporadic investigations report on the role of methylation in gene body regions for gene activity, there were at least some studies that documented the suppression of gene expression and heterochromatinization, due to coding region methylation [33,34]. 

Understanding the mechanisms for recognition and uptake of extracellular nucleic acids by plants has only begun in recent years [35]. Several studies documented active plant perception of extra-celluar RNAs originating from plant viruses and pathogenic bacteria and acting as genuine pathogen-associated molecular patterns (PAMPs) and microbe-associated molecular patterns (MAMPs) to induce the pattern-triggered plant immune signaling [36,37]. MAMPs and PAMPs are recognized by pattern recognition receptors (PRRs). Plant PRRs are either, surface-localized receptor kinases, or receptor-like proteins, containing various ligand-binding ectodomains [38]. The molecular mechanisms, and the specific PRRs responsible for extracellular nucleic acid recognition and uptake, are generally uncharacterized. Niehl et al. [37] showed that exogenously applied dsRNAs act in plants via a somatic embryogenesis receptor-like kinase 1 (SERK1), and not through antiviral dicer-like (DCL) proteins, and suggested SERK1 as a potential dsRNA receptor. It is likely that transgene-encoding dsRNAs applied in this study and dsRNAs used in other related studies [5,6,9,10,11,12,13,39] could also act via PRRs. Further research is needed to shed light on the mechanisms of extracellular dsRNA uptake, local and systemic dsRNA movements, and processing in plant tissues and cells. It is possible that exogenously applied dsRNA molecules could also affect transcript levels of plant endogenous genes related to various signaling pathways. Active studies in this direction could promote development of new alternatives to genetically modified crops and new appealing methodologies that can be used in plant functional studies. 

## 4. Materials and Methods 

### 4.1. Generation of Transgenic Arabidopsis

Plants (*Arabidopsis thaliana* ecotype Columbia L.) were grown in pots filled with commercially available rich soil in a controlled environmental chamber at 22 °C (Sanyo MLR-352, Panasonic, Osaka, Japan), kept on a 16/8 h day/night cycle at a light intensity of ~120 μmol m^−2^ s^−1^.

To generate the *EGFP*- and *NPTII*-overexpressing plants, we used the binary plasmid construction pZP-RCS2-*EGFP*-*NPTII* (Figure 1a) that was kindly provided by Professor Alexander Krichevsky (State University of New York, Stony Brook, NY, USA) [24]. The construction carried the *NPTII* and *EGFP* genes under the control of the double CaMV 35S promoters. *A. thaliana* was transformed by floral-dip as described [25]. The transgenic lines used in this study were T_3_ homozygous plants with single copy insertion.

### 4.2. dsRNA Synthesis and Application

dsRNA of *EGFP* and *NPTII* was synthesized using MEGAscript RNAi Kit (ThermoFisher Scientific, Waltham, MA, USA). The T7 promoter sequence was introduced into both the 5’ and 3’ ends of *EGFP* and *NPTII* by PCR. The primers are shown in Table S1 and Figure 1a. The obtained PCR products were used as templates for *in vitro* transcription and dsRNA synthesis following the manufacturer’s protocol. The resultant dsRNAs were analyzed by gel electrophoresis and were spectrophotometrically analyzed to estimate the amount, purity, and integrity of the dsRNAs. The dsRNAs were applied on the surface of four-week-old *Arabidopsis* by spreading with individual soft brushes (natural pony hair) sterilized by autoclaving. We did not observe wounding visually and using confocal microscopy. All the treatments were performed at 9:00–9:30 pm. 

### 4.3. Total RNA Extraction, Reverse Transcription, and qRT-PCR

The leaves of *A. thaliana* for nucleic acid isolation were collected from the same four-week-old individual plant at all time points (before, 1, 7 and 14 days post-treatments) for each type of treatment. One typical adult leaf was used for the nucleic acid isolation each time. RNA isolation was carried out from the *A. thaliana* leaves before treatment, 1, 7, and 14 days post-treatments. The isolation of total RNA was performed using the cetyltrimethylammonium bromide (CTAB)-based protocol and complementary DNAs were synthesized as described [40]. The reverse transcription products were amplified by PCR and verified on the absence for DNA contamination using primers listed in Table S1. The qRT-PCRs were performed with EvaGreen Real-time PCR (Biotium, Hayward, CA, USA) as described in [25], using cDNAs of *NPTII*, *EGFP*, and two internal controls (*GAPDH* and *UBQ*) selected in previous studies as relevant reference genes for qRT-PCRs on *Arabidopsis* [41]. The expression was calculated by the 2^−ΔΔCT^ method [42]. All GenBank accession numbers and primers are listed in Table S1. 

### 4.4. Laser Scanning Microscopy

The leaves were mounted in distilled water in a Petri dish and were observed under a Zeiss LSM 780 confocal microscope (Far East Centre of Electron Microscopy, National Scientific Center of Marine Biology, Far Eastern Branch of the Russian Academy of Sciences, Vladivostok, Russia) equipped with a Plan-Apochromat 20×/0.8 objective. The excitation wavelength of an argon laser was set at 488 nm and *EGFP* emission was detected using a 520–525 nm band pass filter. To quantify *EGFP* fluorescence 10 Z-stacks were captured for each leaf (5 Z-stacks from the top and 5 Z-stacks from bottom side of the leaf). For fluorescence intensity quantification, Z-stacks were processed using ImageJ 1.51w image processing software [43,44]. Each Z-stack was imported into ImageJ and z-projected using sum projection. The projected images reached the threshold using the method from Otsu [45], and the area and mean fluorescence of the foreground (signal of *EGFP*) and the background along the entire image were measured. The corrected *EGFP* fluorescence was calculated according to the formula: Corrected *EGFP* fluorescence = mean fluorescence of the foreground − (area of the foreground × mean fluorescence of the background).

### 4.5. Protein Extraction and Western Blotting

Proteins were isolated from *A. thaliana* by grinding 30 mg of frozen leaf tissue in 300 µL of 0.1 M sodium phosphate buffer pH 8.0 [46] with 4% sodium dodecyl sulfate, followed by ultrasound treatment for 30 s at maximum amplitude, using Q55 Qsonica sonicator (Qsonica LLC, Newtown, CT, USA). After centrifugation (16,000× *g* for 20 min at +4 °C), the protein concentration was quantified using Qubit^®^Protein Assay Kit and Qubit 2.0 fluorometer (Invitrogen, Carlsbad, CA, USA). A quantity of 5 µg of the protein extracts were separated by SDS–PAGE in 12% polyacrylamide gels using a Helicon VE-10 (Helicon, Moscow, Russia) at 200 V and were electro-transferred to polyvinylidene difluoride (PVDF) membrane. After blocking for 1 h in PBS (1.7 mM KH_2_PO_4_, 5.2 mM Na_2_HPO_4_, 150 mM NaCl) with 2.5% nonfat dry milk at room temperature, membranes were incubated overnight at +5 °C with anti-GFP antibody (ab6556 Abcam, Cambridge, United Kingdom, USA) at 1:1000 dilution. Following three washings with PBS containing 0.1% Tween 20 and 2.5% nonfat dry milk at room temperature, the membrane was incubated with secondary antibody (ab6112 Abcam, USA) at 1:2000 dilution for 1 h. After three washings, the membranes were visualized by treatment with 40 mL water solution containing 10 mg 3,3′-diaminobenzidine, 4 mL EtOH, 2 mL of 1 M Tris-HCl (pH 7.6), and 25 µL H_2_O_2_. As a positive control, we used total proteins purified from *E. coli* Rosetta(DE3)pLysS transformed with pET28a-*EGFP* (Merck, Darmstadt, Germany). The *EGFP* from pZP-RCS2-*EGFP-NPTII* plasmid was PCR-amplified using primers (Table S1) containing BamH I and Hind III restriction sites, The PCR products were digested with the restriction enzymes (SibEnzyme, Novosibirsk, Russia), and the resulting DNA fragments were ligated to pET28a.

### 4.6. DNA Extraction and Bisulfite Sequencing

Sampling of the leaf material for DNA extraction was performed as described above. Extraction of genomic DNA and bisulfite sequencing were performed as described [47]. After DNA conversion, a 352-bp *NPTII* and a 349-bp *EGFP* fragments were amplified using primers listed in Table S1. The level of C to T transitions in the converted PCR products was greater than 95%. We sequenced 18 clones for each DNA region from the 2 biological replicates (9 clones per each individual plant).

### 4.7. Detection of EGFP-siRNA by Stem-Loop RT-PCR and DNA Sequencing

For the analysis, we retrieved *EGFP*-siRNAs from the NCBI siRNA database (Available online: https://www.ncbi.nlm.nih.gov/projects/genome/rnai/) and based our stem-loop primer construction on a EGFP-siRNA (Accession: Pr008640320, Pr120179) with typical structure deposited for the cloning vector pEGFP-N1 (accession U55762), which bears identical *EGFP* coding sequence to pZP-RCS2-*EGFP*-*NPTII* (used in our work). For isolation of low molecular weight (LMW) RNA fraction, we began isolation of total RNA using the CTAB-based protocol as described [40]. After incubation of 1 mL of the water phase, containing total RNA mixed, with 250 µL of 10 M lithium chloride at 4 °C overnight, we separated low-molecular weight (LMW) RNA by centrifugation at 16,000× *g* for 20 min, followed by the collection of the aqueous phase, containing LMW RNAs. Then, the LMW RNA was precipitated as described [48]. The RT reaction (20 µL) was performed with 0.2 µL of *EGFP*-siRNA stem-loop RT primer (100 µM) and ThermoScript reverse transcriptase (Invitrogen, Life Technologies) using the conditions of 16 °C for 30 min, 30 °C for 30 s, 42 °C for 30 s, 50 °C for 1 s for 60 cycles, and 85 °C for 5 min according to published pulsed RT protocol and primer design instructions [26]. The targeted *EGFP*-siRNA was PCR amplified as described [49] with the 25 µL of the PCR mixture containing 0.1 µL of *EGFP*-specific forward and universal reverse primers (100 µM) designed as described [26]. The amplification conditions consisted of one cycle at 95 °C for 2 min followed by 40 cycles of 95 °C for 10 s, 58 for 10 s, and 72 °C for 20 s. The primer sequences are listed in Table S1. The PCR products were isolated from agarose gels using a Cleanup Mini Kit (Eurogene, Moscow, Russia), subcloned, and sequenced as described [49].

## Figures and Tables

**Table 1:** Methylation status in the *NPTII* and *EGFP* transgene protein-coding regions in the transgenic L1 line of *A. thaliana* before and 7 days after foliar application of *NPTII*-dsRNA, *EGFP*-dsRNAs, or filtered sterile water.

Gene	Treatment	CG	CHG	CHH
*NPTII*	Water (before) Water (7 dpa)	65.6 ± 2.1	60.1 ± 3.3	55.9 ± 3.8
		65.3 ± 3.2	63.3 ± 2.4	59.3 ± 4.7
	*NPTII*-dsRNA (before) *NPTII*-dsRNA (7 dpa)	68.5 ± 4.4	60.7 ± 3.0	51.6 ± 4.5
		82.5 ± 4.9 **	79.2 ± 3.4 **	77.5 ± 3.6 **
	*EGFP*-dsRNA (before) *EGFP*-dsRNA (7 dpa)	65.3 ± 3.4	65.0 ± 3.7	55.6 ± 2.4
		80.7 ± 4.3	78.2 ± 2.9	78.1 ± 2.2 **
*EGFP*	Water (before) Water (7 dpa)	62.1 ± 2.4	59.2 ± 3.1	54.7 ± 3.9
		62.4 ± 4.2	59.5 ± 2.8	57.9 ± 5.2
	*NPTII*-dsRNA (before) *NPTII*-dsRNA (7 dpa)	65.2 ± 5.9	62.2 ± 3.3	59.3 ± 4.3
		75.8 ± 5.7	75.9 ± 4.6 *	73.9 ± 3.4 **
	*EGFP*-dsRNA (before) *EGFP*-dsRNA (7 dpa)	59.9 ± 4.2	56.1 ± 3.8	54.1 ± 3.2
		74.9 ± 4.8	75.1 ± 4.7 **	73.1 ± 3.0 **

The data are presented as the mean ± SE, * *P* < 0.05; ** *P* < 0.01 versus the values measured before treatment.

## Supplementary Materials

Supplementary materials can be found at https://www.mdpi.com/1422-0067/20/7/1585/s1. Table S1 Primers used for amplification of the *NPTII* and *EGFP* transgenes in RT-PCR, qRT-PCRs, and bisulfite sequencing. Fig. S1 DNA sequencing of the amplification product including coding sequences of both the *EGFP* and *NPTII* transgenes and their regulatory elements.
