# Supplementary material for: Induction of Transgene Suppression in Plants via External Application of Synthetic dsRNA
Source: Int J Mol Sci. 2019 Mar 29;20(7):1585. doi: 10.3390/ijms20071585 (PMC6479969; doi:10.3390/ijms20071585)
Supplement: Supplementary file 1 [file ijms-20-01585-s001.pdf]

Table S1 Primers used for amplification of the *NPTII* and *EGFP* transgenes in RT-PCR, qRT-PCRs, and bisulfite sequencing

Gene name (GenBank acc. no)	Primer name	Primers, 5′-3′
Primers for cDNA check-up on DNA contamination, 5′-3′		
AtGAPDH<br>(NM_111283)	AtGapdh-s<br>AtGapdh-a	CTG GAA TGT CTT TCC GTG TC<br>ATT CGT TGT CGT ACC ATG AC
Primers for PCR and real-time PCR, 5′-3′		
EGFP<br>(U55762,<br>AY818363)	R1s<br>R1a<br>R2s<br>R2a	CAAGCATTCTACTTCTATTG<br>CTCGCCCTTGCTCACCAT<br>TGCTGCTGCCCGACAACCACTAC<br>CTTGTACAGCTCGTCCATGCC
*npt*II (AY818371)	R3s<br>R3a<br>R4s<br>R4a	TTGCTGAAGAGCTTGGCGGCGAAT<br>TCAGAAGAACTCGTCAAGAAGG<br>CACGACGGGCGTTCCTTGCGC<br>GATACTTTCTCGGCAGGAGCAA
AtGAPDH<br>(NM_111283)	AtGapdh-real-s<br>AtGapdh-real-a	TTG GTG ACA ACA GGT CAA GCA<br>AAA CTT GTC GCT CAA TGC AAT
AtUBQ<br>(NM_001084884)	AtUBQ-realS<br>AtUBQ-realA	GGCCTTGTATAATCCCTGATGAATAAG<br>AAAGAGATAACAGGAACGGAAACATAGT
Specific primers for dsRNA design, 5′-3′		
EGFP<br>(U55762,<br>AY818363)	D1s<br><br>D1a	TAATACGACTCACTATAGGGAGAATGGTGAGCAAGGGC<br>GAGGAG<br>TAATACGACTCACTATAGGGAGATTACTTGTACAGCTCGT<br>CCATG
*npt*II<br>(AY818371)	D2s<br><br>D2a	TAATACGACTCACTATAGGGAGAATGTGGATTGAACAAG<br>ATGGATTG<br>TAATACGACTCACTATAGGGAGATCCACCATGATATTCG<br>GCAAGCAG
Specific primers for pET28a-EGFP design, 5′-3′		
EGFP<br>(U55762,<br>AY818363)		CTA CGG ATC CAT GGT GAG CAA GGG CGA GGA<br>GCA TAA GCT TTT ACT TGT ACA GCT CGT CCA
Primers for bisulfite DNA sequencing, 5′-3′		
EGFP<br>(U55762,<br>AY818363)	M1s<br>M1a	CAC CAT CTT CTT CAA RRA C<br>GTT GGG GTY TTT GYT YAG
*npt*II<br>(AY818371)	M2s<br>M2a	TTC TTT TTR TCA ARA CCR ACC<br>TGA TGC TYT TYG TYY AGA T
Primers for stem-loop RT-PCR, 5′-3′		
EGFP-siRNA<br>(Pr008640320;<br>Pr120179)	stem-loop-RT1	GTTGGCTCTGGTGCAGGGTCCGAGGTATTCGCACCAGAG<br>CCAACAAGTTC
	stem-F1	GCGGCGGGAACGGCATCAAGGT
	universal-R1	GTGCAGGGTCCGAGGT
Primers for detection of the readthrough transcript, 5′-3′		
EGFP (U55762,<br>AY818363)	R2s	TGCTGCTGCCCGACAACCACTAC
*npt*II (AY818371)	R4a	GATACTTTCTCGGCAGGAGCAA

Fig. S1 DNA sequencing of the amplification product including coding sequences of both the *EGFP* and *NPTII* transgenes and their regulatory elements. Highligheted in orange and green are the *EGFP* and *NPTII* coding sequences, respectively; highlighted in brown and blue are the sequences of nopaline synthase terminator and the double CaMV 35S promoter. The positions of primers are underlined.

TGCTGCTGCCCGACAACCACTACCTGAGCACCCAGTCCGCCCTGAGCAAAGACCCCAACGAGAAGCGCGATCACATGGTC
CTGCTGGAGTTCGTGACCGCCGCCGGGATCACTCTCGGCATGGACGAGCTGTACAAGTCCGGACTCAGATCTCGAGCTCAA
GCTTCGAATTCTGCAGTCGACGGTACCGCGGGCCCGGGATCCACCTGATCTAGATCCCGATCGTTCAAACATTTGGCAATA
AAGTTTCTTAAGATTGAATCCTGTTGCCGGTCTTGCGATGATTATCATATAATTTCTGTTGAATTACGTTAAGCATGTAATAA
TTAACATGTAATGCATGACGTTATTTATGAGATGGGTTTTTATGATTAGAGTCCCGCAATTATACATTTAATACGCGATAGA
AAACAAAATATAGCGCGCAAACTAGGATAAATTATCGCGCGCGGTGTCATCTATGTTACTAGATCGGGCGGCCGCACCCA
TAATACCCATAATAGCTGTTTGCCATCGCTACCTTAGGACCGTTATAGTTAACCGGTCAACATGTGGAGCACGACACACTT
GTCTACTCCAAAAATATCAAAGATACAGTCTCAGAAGACCAAAGGGCAATTGAGACTTTTCAACAAAGGGTAATATCCGG
AAACCTCCTCGGATTCCATTGCCCAGCTATCTGTCACTTTATTGTGAAGATAGTGGAAAAGGAAGGTGGCTCCTACAAATG
CCATCATTGCGATAAAGGAAAGGCCATCGTTGAAGATGCCTCTGCCGACAGTGGTCCCAAAGATGGACCCCCACCCACGA
GGAGCATCGTGGAAAAAGAAGACGTTCCAACCACGTCTTCAAAGCAAGTGGATTGATGTGATAACATGGTGGAGCACGAC
ACACTTGTCTACTCCAAAAATATCAAAGATACAGTCTCAGAAGACCAAAGGGCAATTGAGACTTTTCAACAAAGGGTAAT
ATCCGGAAACCTCCTCGGATTCCATTGCCCAGCTATCTGTCACTTTATTGTGAAGATAGTGGAAAAGGAAGGTGGCTCCTA
CAAATGCCATCATTGCGATAAAGGAAAGGCCATCGTTGAAGATGCCTCTGCCGACAGTGGTCCCAAAGATGGACCCCCAC
CCACGAGGAGCATCGTGGAAAAAGAAGACGTTCCAACCACGTCTTCAAAGCAAGTGGATTGATGTGATATCTCCACTGAC
GTAAGGGATGACGCACAATCCCACTATCCTTCGCAAGACCCTTCCTCTATATAAGGAAGTTCATTTCATTTGGAGAGGACG
TCGAGAGTTCTCAACACAACATATACAAAACAAACGAATCTCAAGCAATCAAGCATTCTACTTCTATTGCAGCAATTTAAA
TCATTTCTTTTAAAGCAAAAGCAATTTTCTGAAAATTTTCACCATTTACGAACGATAGCCATGGTCCGGACTCAGATCTCGA
GCTCAAGCTTCGAATTCTGCAGTCGACATGATTGAACAAGATGGATTGCACGCAGGTTCTCCGGCCGCTTGGGTGGAGAGG
CTATTCGGCTATGACTGGGCACAACAGACAATCGGCTGCTCTGATGCCGCCGTGTTCCGGCTGTCAGCGCAGGGGCGCCCG
GTTCTTTTTGTCAAGACCGACCTGTCCGGTGCCCTGAATGAACTGCAGGACGAGGCAGCGCGGCTATCGTGGCTGGCCACG
ACGGGCGTTCCTTGCGCAGCTGTGCTCGACGTTGTCACTGAAGCGGGAAGGGACTGGCTGCTATTGGGCGAAGTGCCGGGG
CAGGATCTCCTGTCATCTCACCTTGCTCCTGCCGAGAAAGTATCCATCATGGCTGATGCAATGCGGCGGCTGCATACGCTTG
ATCCGGCTACCTGCCCATTCGACCACCAAGCGAAACATCGCATCGAGCGAGCACGTACTCGGATGGAAGCCGGTCTTGTC
GATCAGGATGATCTGGACGAAGAGCATCAGGGGCTCGCGCCAGCCGAACTGTTCGCCAGGCTCAAGGCGCGCATGCCCGA
CGGCGATGATCTCGTCGTGACCCATGGCGATGCCTGCTTGCCGAATATCATGGTGGAAAATGGCCGCTTTTCTGGATTCATC
GACTGTGGCCGGCTGGGTGTGGCGGACCGCTATCAGGACATAGCGTTGGCTACCCGTGATATTGCTGAAGAGCTTGGCGGC
GAATGGGCTGACCGCTTCCTCGTGCTTTACGGTATCGCCGCTCCCGATTCGCAGCGCATCGCCTTCTATCGCCTTCTTGACG
AGTTCTTCTGA
